# Bringing Green Homes within Reach: Healthier Housing for More People

**DOI:** 10.1289/ehp.116-a24

**Published:** 2008-01

**Authors:** Charles W. Schmidt

Gaze upon the Helliers’ half-built house in Bristol, Vermont, and you might think you’re looking at an ordinary home construction project. Table saws, building materials, and piles of earth lie around the newly framed dwelling, while a crew of carpenters mills around the site, dressed for warmth in the chilly fall air. But look closer, and some unique features emerge. The exterior frame is wrapped in an outer layer of heat-trapping insulation. Sunshine streams in through large, south-facing windows, flooding the interior living spaces with light. Once the house is completed, solar panels will supply the family’s hot water and much of its electrical power. And indoor finishes, paints, rugs, and fabrics will be nontoxic.

In short, the Helliers’ house is being built to be green. And that puts it in good company; new green homes jumped in number by 30% between 2005 and 2006 and could include up to 5% of the entire U.S. housing market within five years, predicts McGraw-Hill Construction, an industry information provider, in its June 2006 *Residential Green Building SmartMarket Report*. That makes green homes bright spots in an otherwise dismal housing market facing its worst slump in decades.

To everyone’s benefit, green homes link sustainable materials and practices with better human and environmental health. “You’re really looking at a tripod of components,” says David Johnston, president of green building consultancy What’s Working and author of *Green from the Ground Up*, a forthcoming book on sustainable residential design. “First, energy efficiency has to be above minimal code requirements for your climate. The second component has to do with improved water and resource efficiency, and the third concerns indoor air quality. If your design doesn’t address all three of these issues, then you don’t have a green home.”

According to the U.S. Environmental Protection Agency (EPA) Office of Air and Radiation, indoor air is typically 2–5 times more polluted than outdoor air, owing to the presence of asthma-inducing agents such as mold and toxic chemicals in carpets, paints, and other synthetic materials. In fact, the EPA ranks indoor air as one of the top five human health risks, says agency spokesperson Dave Ryan. By requiring nontoxic materials, green designs limit indoor exposure to carcinogens such as formaldehyde in manufactured wood products including sheathing and particleboard, and to volatile organic compounds (VOCs) in finishes.

Home energy uses also contribute to global warming. The Energy Information Administration (EIA) in Washington, DC, estimates that domestic power demands account for 21% of all the greenhouse gases emitted in the United States. The construction industry as a whole accounts for 48% of U.S. greenhouse gas emissions, according to advocacy group Architecture 2030. And by optimizing insulation, green designs save on oil and gas bills, which are (quite literally in poorly insulated homes) going through the roof.

But even as green homes gain in popularity, they’re also dogged by a sticky association with the rich, seen by many as too pricey for ordinary buyers. Indeed, McGraw-Hill Construction identified cost perceptions as a top obstacle to green building among homeowners and builders alike. Fueling that preoccupation with cost is a media obsession with “eco-mansions,” laments Charles Lockwood, a green building consultant in Los Angeles and New York. “Most of what you see in the press would leave you thinking you’d have to live in Malibu or Aspen to afford one of these places,” he says. To wit: the *Maine Sunday Telegram* in Portland ran a feature on 4 November 2007 titled “Unaffordably Green?” about a $1 million ultra-green home in nearby Freeport that was unsold after a year on the market.

Christopher Briley, an architect with Green Design Studio in Yarmouth, Maine, concedes that most people who build their own houses have above-average incomes (McGraw-Hill Construction’s *SmartMarket Report* shows that nearly two-thirds of those who buy green homes make more than $50,000 a year). And buyers with more money to spend, Briley says, are apt to mix green design elements in with a host of other more expensive features—radiant floors and granite countertops, for instance—that skew costs higher. “A lot of these houses are going to be expensive anyway,” he says. “But that doesn’t mean you can’t have an affordable green house. It’s all about where you decide to spend your money.”

## A Growing Trend

National standards for green homes are just now emerging. Energy Star, a joint program by the EPA and the Department of Energy (DOE), has been setting energy sustainability targets for lighting, appliances, and home electronics since the early 1990s. But being limited to energy, the program addresses only one component of green design.

The newest and farthest-reaching national standards have come from the U.S. Green Building Council (USGBC). Since 2000, the USGBC’s Leadership in Energy and Environmental Design (LEED) system has set the bar for sustainability in commercial settings. Today, roughly 5% of public buildings in the United States are LEED-certified. Now, with the organization’s LEED for Homes program, which was launched officially in November 2007 after a two-year pilot project, the council is poised to issue comprehensive national guidelines for residential green design. The system requires third-party verification by inspectors who qualify homes as basic-, silver-, gold-, or platinum-certified, depending on how many green features they have. As this article was going to press, 381 homes nationwide had achieved some type of LEED status, and 10,000 more were in the pipeline, according to USGBC spokesperson Ashley Katz.

The National Association of Home Builders (NAHB) in Washington, DC, also offers guidance to builders who are going green. The association released its *Model Green Home Building Guidelines* in 2006 and is set to launch a Green Professional certification program in 2008. Certification will be awarded after 24 hours of course work and requires builders to maintain regular additional continuing education credits. The NAHB has also initiated a process, along with the International Code Council, to develop a voluntary standard for green home building construction practices to be compliant with the American National Standards Institute. The standard is expected to be in place by the end of 2008.

## Banking on Low-Hanging Fruit

For the USGBC and others in the green building community, changing cost perceptions has become a top priority. Steve Konstantino, who runs Maine Green Building Supply in Portland, says a wealth of cost-effective options are available to consumers. For instance, he says, PaperStone™ countertops made with recycled paper and water-based resins cost roughly the same as petroleum-based Corian^®^. Bamboo hardwoods can be sustainably cultivated, but the plants aren’t locally grown in the United States outside of California. In fact, most of the bamboo sold worldwide comes from Asia. The best option, Konstantino advises, is to go with local hardwoods from sustainably harvested forests. Costs begin to climb when buyers ask for reclaimed woods harvested from old barns and industrial buildings. These materials figure prominently in many green home standards, but they also cost upwards of $7 per square foot and typically more, compared with other options such as pine, which generally costs $5 per square foot or less.

To get the most bang for the buck, cash outlays in green building should target low-hanging fruit that can deliver the bulk of a home’s sustainability for the lowest price, Briley says. Merely siting a house so that the longest walls and largest windows face south (in the Northern Hemisphere) is the single most important thing a builder can do to keep homes naturally warm in colder climates, he adds. South-facing orientations optimize solar exposure as the sun travels across the sky. According to calculations by the Rocky Mountain Institute, an environmental think tank in Boulder, Colorado, pointing a house in the right direction can shave 30% off monthly utility bills. “That’s free light and heat,” Briley says. “I’m amazed at how many homes are oriented toward the road without giving a single thought to the sun.” In warmer climates, of course, such a strategy would drastically increase cooling needs during the summer; appropriate siting strategies would therefore include the use of increased natural shading.

The next bunch of low-hanging fruit is home sealing and insulation. Most homes built after World War II—when many assumed that heating oil would stay cheap forever—were barely insulated at all. After the oil shocks of the 1970s, builders began adding more insulation, but green designs go a step further; they aim to make living spaces virtually airtight. If done correctly, interiors are so tightly sealed that, with doors and windows closed, mechanical ventilators must be used for air exchange with the outdoors. The insulating process starts in the frame—liberal amounts of caulk seal interior spaces between stacked wall studs, while hard-drying urethane foam gets squeezed into every nook and cranny that could produce a draft.

Most conventional homes limit insulation to the inside of the frame; green homes also wrap the frame’s exterior to create what’s called a “thermal break.” That’s a critical step, Briley asserts. “If you insulate only between wall studs that you cover with [wallboard], that means you haven’t insulated any of the points where the studs meet the exterior wall. The consequence is that up to twenty percent of the house isn’t really insulated at all. Providing a thermal break, or insulating around your studs, will maximize your coverage.”

Custom woodworker Skimmer Hellier, who owns and designed the green home described earlier, cautions that with an airtight interior, it’s important to avoid toxic materials indoors. “If you’re building a super-insulated house and you put in some type of synthetic wall-to-wall carpeting, you could be dealing with some troublesome air quality issues,” he says. “You’ve got to avoid formaldehyde or VOCs in paints—offgassing is something you’ve got to pay attention to.” But ultimately, airtight homes are healthier for their inhabitants, Hellier adds, in part because they block drafts where moist air gets into the wall cavity and condenses at dew points inside the wall—a chief cause of mold growth behind wallboard. (It’s important to note that other steps, for instance, tight roofing and overhangs to prevent water intrusion, in addition to appropriately placed vapor barriers in wall structures, can also block mold growth.)

Briley adds that passive solar design can be augmented by incorporating thermal mass inside the house, using heavy, dense materials such as concrete slabs under floor-boards or masonry fireplaces in sunny rooms. These structures stabilize interior temperatures and take the spikes out of heating and cooling, he says.

## Thinking about Cost

By creating an airtight building envelope, homeowners can lower heating and cooling costs by 50% or more. The up-front expense of doing so can be minimal, adds Greg Kats, managing director of Good Energies, a venture capital firm that invests in sustainable technology. Kats’s investigations have revealed the average “green premium” for sustainable design totals no more than $3–5 per square foot, generally to cover added insulation, double- or triple-glazed windows, high-efficiency appliances, and in some cases, a builder’s learning curve.

But the premium dwindles as builders gain experience and professional contacts in the field, Johnston says. “We’ve found that the first time builders build green, the houses run about ten percent higher,” he explains. “But then they get their materials and subcontractors figured out, so that the second house costs three percent more and by the time they get to the third house, costs aren’t any more than one percent higher.”

The main thing to consider with green design is positive monthly cash flow, Johnston emphasizes. “If savings from energy conservation are greater than the increase in the monthly cost of the mortgage or construction loan, then the homeowner is literally making money month after month,” he says. “Energy conservation is not a cost, it’s an investment that only gets more valuable over time. That holds true no matter the income bracket of the homeowner.” Although payback over time is a certainty, the length of time it will take is almost impossible to predict because of uncertainty about future energy prices, says Johnston. The question is how much buyers will invest up front for savings down the line.

Passive solar technologies, sufficient insulation, high-efficiency fixtures and appliances, and sustainable building materials yield the most bang for the buck by far, says Hellier. Compact fluorescent lamps (CFLs), for example, cost 3–4 times more than incandescent bulbs, but they use a fraction of the energy and can last ten times longer. After that, systems become far more expensive with diminishing returns. Solar power is a case in point: Solar hot water units, which cost from $4,000 to $9,000 installed depending on size requirements (in contrast to $700 or less for a conventional unit), have a typical payback of five years or less, making them somewhat affordable. But solar photovoltaic (PV) panels, which supply a home’s electricity, can run $6,000 per kilowatt or more installed (most U.S. homes need between 2 and 5 kilowatts of capacity to accommodate all their electrical needs throughout the year). Similarly, the cost of wind energy—best for rural properties with at least an acre of land—runs between $3,000 and $5,000 per kilowatt installed.

Fortunately, a host of rebate programs can offset some of this expense. By tapping a mix of these programs, the Helliers cut costs for their own 3.5-kilowatt PV installation—and their solar hot water heater—by about a third. And after that initial investment, solar and wind energy are free. Ideally, excess renewable power generated by those systems during sunny (or windy) days can be dumped back into the local electrical grid if the home is connected to it. Then, if utilities use “net metering,” the retail value of that electricity can be deducted from what homeowners pay for power on wind-free or cloudy days. [For more on incentive programs, see “Room to Grow: Incentives Boost Energy-Efficient Home-building,” p. A32 this issue.]

Yet even with those savings, the pay-back on sun or wind power can take many years, even decades, putting them out of reach of most consumers. One of the main factors driving the expense of solar and wind technology is limited manufacturing, says Cécile Warner, a principal engineer at the DOE’s National Renewable Energy Laboratory in Golden, Colorado. Therefore, as more of these units are sold, prices are expected to fall. So in a sense, those who do buy into these systems are performing a kind of civic duty.

Perhaps the simplest way to build an inexpensive green home is to build a smaller home—a strategy emphasized by LEED for Homes. “Given the inflated sizes of many new U.S. homes, this strategy is a no-brainer,” says Tristan Korthals Altes, managing editor for *Environmental Building News.* In 2006 the average new U.S. single-family home measured 2,459 square feet, according to Gopal Ahluwalia, vice president for research at the NAHB, speaking at that group’s 2007 International Builders’ Show. In 1973, new homes averaged about 1,500 square feet.

Even as green housing embraces affordability, others are trying to bring green principles to affordable housing for low-income populations, including the elderly. A top organization working in this area is Enterprise Community Partners, a Columbia, Maryland–based provider of capital and expertise for developing affordable housing. Through its Green Communities Initiative launched in 2004, the organization has already spent $450 million to build 11,000 green homes within 245 multifamily housing developments in approximately 25 states.

Dana Bourland, who heads the Green Communities Initiative, says Enterprise developed standards through its own “green communities criteria,” which were based on input from several expert organizations in health, planning, and architecture. The standards closely mirror those found in the LEED for Homes program, she adds. “Low-income families in some ways have the most to gain from healthier homes,” she says. “Too many of them live in substandard housing that heightens risks for asthma and other respiratory illnesses. We’ve got anecdotal evidence of immediate health improvements from moving to green residential settings. And what’s more, green homes allow low-income residents to save three to four hundred dollars a year on energy and water bills. So, Enterprise has committed itself to make all its projects green from here on out.”

## Looking to the Future

Moving forward, green homes stand to become far more innovative than they are now. Today, the race is on to make houses that generate all their power from renewable sources affordable to ordinary consumers. Energy regulators in California recently pledged that all new homes built there after 2020 would produce as much energy as they consume, a feature known as “net-zero” energy consumption. The entire country of England plans to make a similar policy mandatory by 2016 in a bid to reduce that nation’s carbon dioxide releases by 60% over 1990 levels by 2050. The first such British home was unveiled in June 2007; it features solar panels, a biomass boiler that burns woodchips, and the capacity to harvest and use rainwater. The architects who designed the house assume the carbon dioxide given off by the boiler is offset by the amounts absorbed when the crop fuel was grown. The home’s cost comes in at roughly 40% more than a conventional house of the same size, concedes its designer, Alan Shingler of Sheppard Robson architects in London. But those costs should fall as more similar homes are built, he adds.

According to Warner, the DOE hopes to make the cost of solar power competitive with grid electricity by 2015 as part of its Building America program, which aims to reduce whole-house energy use in new homes by 50% by 2015 and by 90% by 2020. “That’s an aggressive path we’ve taken hand-in-hand with industry,” she says. Doing that won’t be easy: PV panels—made from crystalline silica—are made using the same painstaking processes used to make semiconductors for the computer chip industry. PV costs, Warner explains, correlate directly with panel size. Therefore, the best way to reduce price is by modifying materials to make them thinner and more efficient in terms of converting light to power. Once that’s achieved, it is conceivable that solar panels, currently limited mostly to rooftops and backyard installations, could wind up in unexpected places such as window shades, awnings—anywhere the sun shines.

Driven by net zero goals, green home research has become striking in its complexity. For instance, NREL principal engineer Craig Christensen works with a software tool called “BEopt” that looks for optimal combinations of 300 different measures pertaining to a building’s outer shell, its interior envelope, equipment, appliances, and more. The software runs hour-by-hour simulations combined with a year’s worth of hourly weather data, looking to compare energy costs versus energy savings. “It helps us set realistic targets,” Christensen explains, referring to goals set by the DOE Building America program.

These are ambitious goals for a country of builders trying to adapt to the green mindset. Some are resistant to change—particularly if they’ve been building a certain way for a long time—and resent the intrusion. Others have already been building green for years, but just haven’t called it that. Still others are starting their careers as certified green builders. “All we need is more people to transform the market place and educate the public,” Briley says. “It will just serve to make us and our buildings better.”

## Green Building Resources

### Building America

http://www.eere.energy.gov/buildings/building_america/

Building America is a public–private partnership sponsored by the U.S. Department of Energy that assembles segments of the building industry that traditionally work independently of one another. The program focuses on developing energy-efficient solutions for new and existing housing that can be implemented on a production basis.

### Database of State Incentives for Renewables and Efficiency

http://www.dsireusa.org/

The Interstate Renewable Energy Council and the North Carolina Solar Center have teamed up to develop the Database of State Incentives for Renewables and Efficiency. This website features clickable maps that allow visitors to access a wide variety of resources on federal and state programs that support purchasing energy from renewable sources.

### Energy Star

http://www.energystar.gov/

Since the early 1990s the federal Energy Star program has helped consumers identify energy-efficient goods and building products. Today, entire buildings can qualify for the Energy Star label. The Energy Star website helps individuals and businesses find products to help them make their homes and workplaces greener.

### Rocky Mountain Institute

http://www.rmi.org/

The Rocky Mountain Institute aims to help everyone from governments to individuals reduce their environmental impact in the most cost-effective manner. The Buildings section on its website offers practical tips on saving energy and money in households and presents a look at the institute’s Built Environment Team, which provides consulting services to developers, architects, and other real estate professionals to help them incorporate cutting-edge efficiency processes in their projects.

### U.S. Green Building Council

http://www.usgbc.org/

The U.S. Green Building Council (USGBC) oversees the Leadership in Energy and Environmental Design program, which rates buildings according to the incorporation of sustainable practices. In addition to information on this program, the USGBC website also provides a state-by-state list of green architects and builders as well as a wealth of educational information on green design, construction, and operations.

## Figures and Tables

**Figure:**
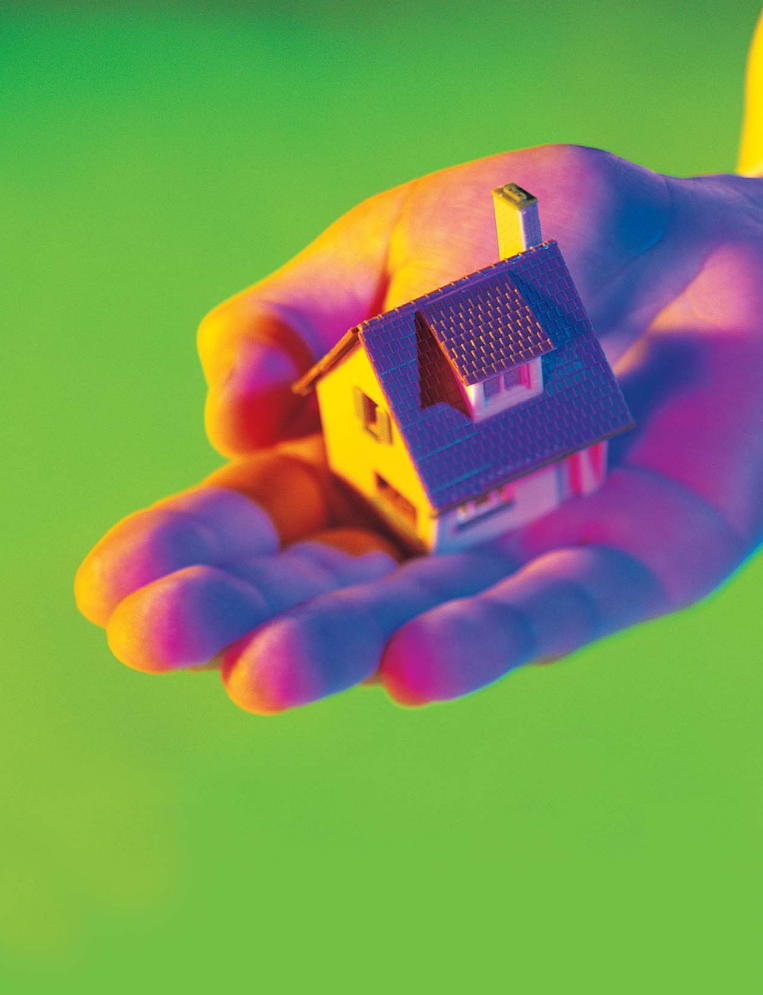


**Figure:**
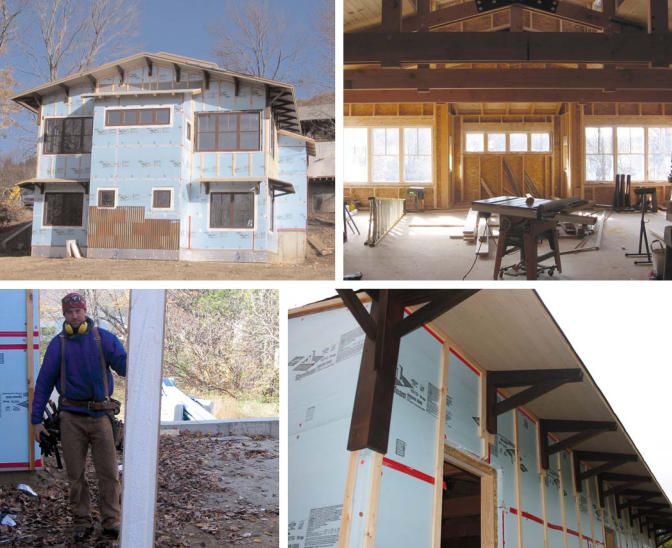
The Hellier home in Bristol, Vermont, is going up green Among other features, a south-facing orientation (top left and right) capitalizes on natural light and thermal energy, while insulated panels (bottom left) and wrapped framing (bottom right) seal the home. This home also enjoys the advantage of know-how: with experience, builders generally can trim much of the “green premium” from such projects.

**Figure:**
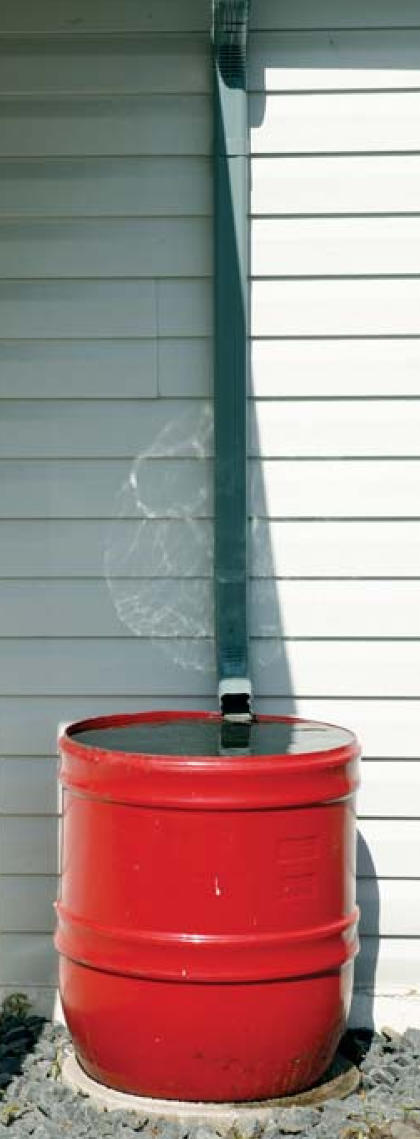
Rainwater collection Rain-water collected from the roof in barrels or cisterns can be used for irrigation and other nondrinking uses.

**Figure:**
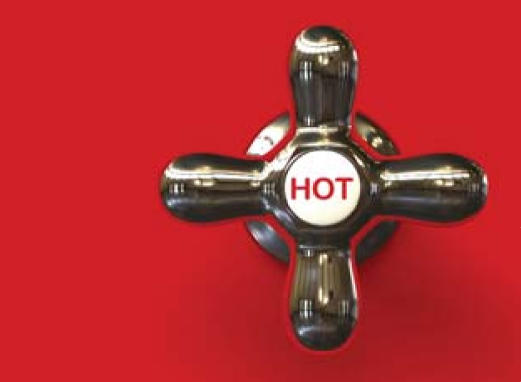
Tankless water heater Tankless water heaters provide hot water only as needed, making them up to a third more energy efficient than conventional water heaters.

**Figure:**
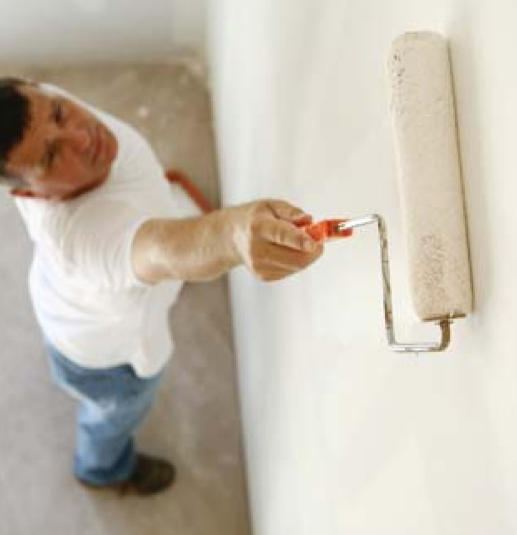
Paint Low- and no-VOC paints and finishes offgas significantly less than conventional products.

**Figure:**
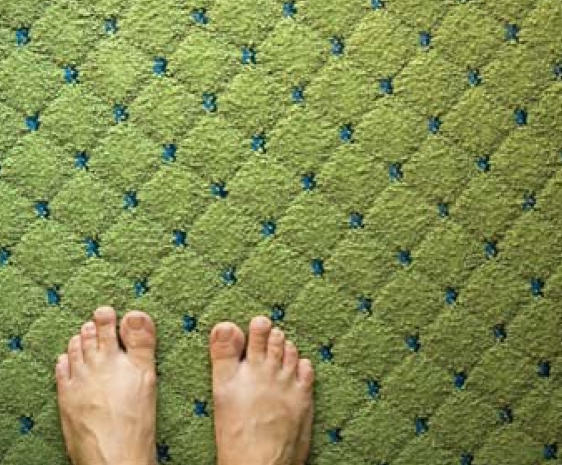
Carpet Low-pile and natural-fiber carpeting traps fewer allergens. Installing carpet with tacks instead of glue also reduces indoor air concentrations of VOCs.

**Figure:**
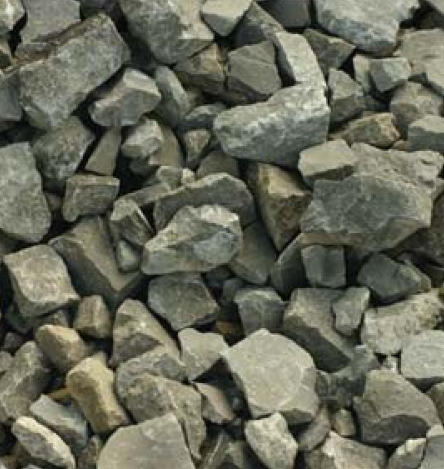
Porous pavements Uncompacted gravel, crushed stone, and open paving blocks reduce or eliminate runoff and allow rain-water to filter into the ground.

**Figure:**
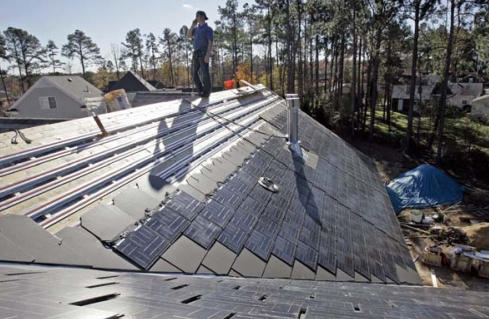
PV shingles Thin-film PV shingles are designed to blend in with regular asphalt shingles.

**Figure:**
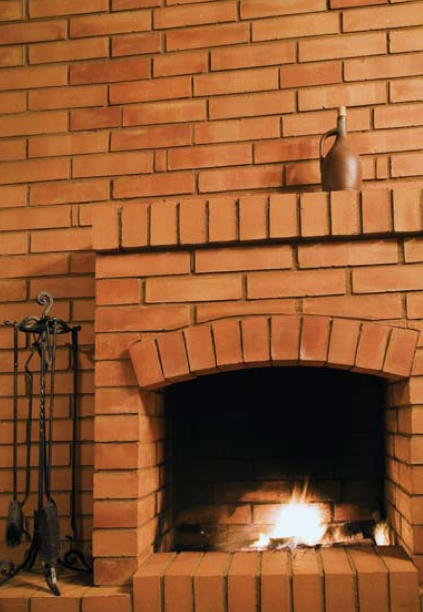
Masonry Stone or brick fireplaces and walls stabilize interior temperatures.

**Figure:**
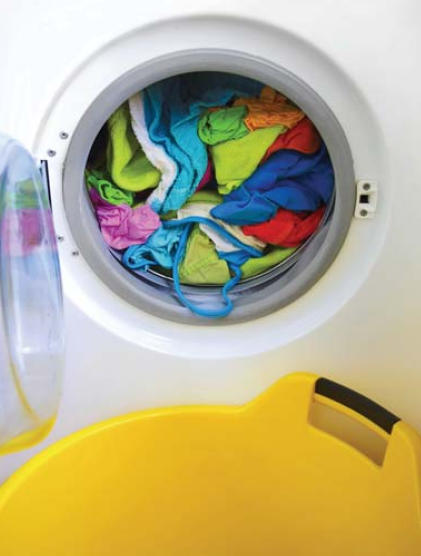
High-efficiency appliances Besides using less energy, high-efficiency laundry equipment uses less water, while high-efficiency refrigerators allow better temperature control.

**Figure:**
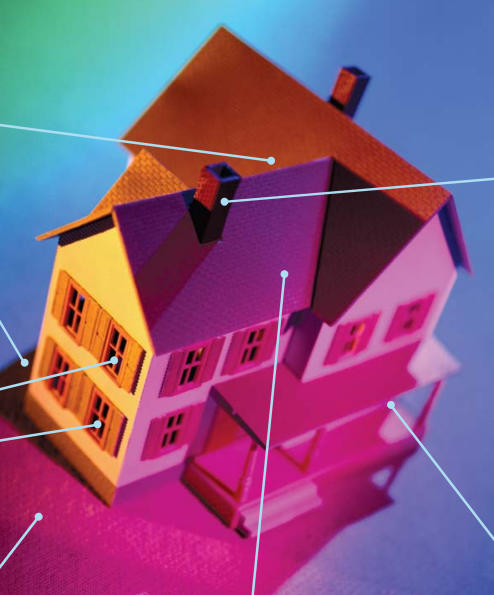
A Few Ways to Make a House Green . . .

